# A Multi-Country Analysis of Prevalence of Anxiety-Induced Sleep Disturbance and Its Associated Factors among In-School Adolescents in Sub-Saharan Africa Using the Global School-Based Health Survey

**DOI:** 10.3390/healthcare9020234

**Published:** 2021-02-22

**Authors:** Bright Opoku Ahinkorah, Richard Gyan Aboagye, Francis Arthur-Holmes, John Elvis Hagan, Joshua Okyere, Eugene Budu, Robert Kokou Dowou, Collins Adu, Abdul-Aziz Seidu

**Affiliations:** 1 School of Public Health, Faculty of Health, University of Technology Sydney, Sydney, NSW 2007, Australia; brightahinkorah@gmail.com; 2 School of Public Health, University of Health and Allied Sciences, Ho PMB 31, Ghana; raboagye18@sph.uhas.edu.gh (R.G.A.); roberthusdowou@gmail.com (R.K.D.); 3 Department of Sociology and Social Policy, Lingnan University, 8 Castle Peak Road, Tuen Mun, Hong Kong, China; frarthur88@gmail.com; 4 Department of Health, Physical Education, and Recreation, University of Cape Coast, Cape Coast PMB, Ghana; 5 Neurocognition and Action-Biomechanics-Research Group, Faculty of Psychology and Sport Sciences, Bielefeld University, Postfach 10 01 31, 33501 Bielefeld, Germany; 6 Department of Population and Health, University of Cape Coast, Cape Coast PMB TF0494, Ghana; joshuaokyere54@gmail.com (J.O.); budueugene@gmail.com (E.B.); abdul-aziz.seidu@stu.ucc.edu.gh (A.-A.S.); 7 Department of Health Promotion, Education and Disability Studies, Kwame Nkrumah University of Science and Technology, Kumasi PMB AK, Ghana; collinsadu80@yahoo.com; 8 College of Public Health, Medical and Veterinary Services, James Cook University, Townsville, QLD 4811, Australia

**Keywords:** anxiety-induced sleep disturbance, in-school adolescents, mental health, rational emotive behavioral education, social emotional learning, sub-Saharan Africa

## Abstract

(1) Background: Among the health problems affecting adolescents, anxiety disorders are considered among the health-compromising or debilitating outcomes that affect adolescents’ mental health. We examined the prevalence and factors associated with anxiety-induced sleep disturbance among in-school adolescents in sub-Saharan Africa (SSA). (2) Methods: This study involved a cross-sectional analysis of data from the Global School-Based Health Survey (GSHS). We analyzed data on 25,454 in-school adolescents from eleven (11) countries in SSA with a dataset between 2010 and 2017. Two multivariable logistic regression models were built to determine the strength of the association between anxiety-induced sleep disturbance and the explanatory variables. The results of the regression analyses were presented using adjusted odds ratios (aOR) and their respective 95% confidence intervals (CIs). Statistical significance was set at *p*-value < 0.05. (3) Results: The overall prevalence of anxiety-induced sleep disturbance among in-school adolescents in SSA was 12.2%. The prevalence ranged from 5.1% in Tanzania to 20.5% in Benin. The odds of anxiety-induced sleep disturbance was higher among adolescents aged 15 and above [aOR = 1.26, 95% CI = 1.15, 1.39] compared to those aged 14 or younger. Additionally, the odds of anxiety-induced sleep disturbance was higher among adolescents who were bullied [aOR = 1.54, 95% CI = 1.42, 1.67], those that felt lonely [aOR = 3.85, 95% CI = 3.52, 4.22], those who had suicidal ideations [aOR = 1.70, 95% CI = 1.52, 1.90], those who had suicidal plan [aOR = 1.26, 95% CI = 1.13, 1.41], those who have had suicidal attempt [aOR = 1.21, 95% CI = 1.08, 1.35], those who used marijuana [aOR = 1.27, 95% CI = 1.06, 1.52], and those who were truant at school [aOR = 1.33, 95% CI = 1.22, 1.46]. However, male adolescents had lower odds of anxiety-induced sleep disturbance [aOR = 0.88, 95% CI = 0.81, 0.95], compared to their female counterparts. (4) Conclusions: We found a relatively high prevalence of anxiety-induced sleep disturbance among in-school adolescents in SSA. Higher age, being female, being bullied, loneliness, having suicidal ideations/plan/attempt, use of marijuana and truancy were risk factors for anxiety-induced sleep disturbance. The findings, therefore, highlight the urgency for policies (e.g., early school-based screening) and interventions (e.g., Rational Emotive Behavioral Education (REBE), Social Emotional Learning (SEL) that target in-school adolescents within the most at-risk populations of anxiety-induced sleep disturbance in SSA.

## 1. Background

According to scholars, anxiety disorders are a set of mental health conditions that are characterized by emotional or social loneliness, worry, sleep disturbance, and excessive fear [1,2,3,4]. Difficulties falling asleep and awakenings during nights are caused by anxiety disorders, and these disorders (anxiety and sleep) are bi-directionally associated, suggesting that each contributes to the development and are a consequence of one another [5,6,7].

Among the health problems affecting adolescents, anxiety-induced sleep disturbance is considered as one that affects their mental health. Empirical findings show that “mental health conditions account for 16% of the global burden of disease and injury in people aged 10–19” [8]. In relation to this, Kessler et al. [9] indicated that about 10–20% of adolescents worldwide experience mental health conditions and half of all mental health issues begin at the age of 14 years.

Anxiety is the ninth leading cause of illness and disability for adolescent aged 15–19 and sixth for those aged 10–14 [8]. In sub-Saharan Africa (SSA), depression and anxiety have been identified as the most common mental disorders [10,11]. From the dominant literature, anxiety disorders among adolescents have been defined and differentiated through various forms: generalized anxiety disorder, separation anxiety disorder, panic disorder, social phobia, and specific phobia [3,12].

The professional and social aspects of life of individuals who experience anxiety disorders are affected because of a deterioration of general condition with a decrease in cognitive behavior and intellectual abilities [13]. For adolescents, symptoms of anxiety disorders can make them engage in risky behaviors (e.g., fights, and stealing, tobacco use, and consumption of illicit drugs (e.g., cocaine, marijuana [1,2]). For in-school adolescents, anxiety disorders are associated with impairment of memory and cognitive functions, and can contribute to poor school performance and academic failure, which can, in turn, lead to further psychiatric disturbances [14]. Hence, understanding the prevalence of anxiety-induced sleep disturbance among in-school adolescents and its associated factors is crucial to education directors and other key stakeholders to design specific interventions at the school settings to reduce anxiety.

Few studies on a global scale have shown the prevalence and correlates of anxiety-induced sleep disturbance among in-school adolescents (e.g., [4,15,16,17,18]). For example, 4.7% of participants in Khan and Khan’s study [1] reported anxiety-induced sleep disturbance among adolescents in Bangladesh. Abbo et al. [16], in their study in Uganda, found a 26.6% prevalence of anxiety-induced sleep disturbance among children aged 3–19. Additionally, in Nigeria, Adewuya et al. [17] found 15% prevalence rates of anxiety-induced sleep disturbance among in-school adolescents aged 13–18. The variations in prevalence of anxiety-induced sleep disturbance in these studies could be attributed to different study settings and the target population of adolescents considered in each study. In addition, studies have shown the factors that trigger anxiety-induced sleep disturbance among adolescents vary across countries and populations. Based on country-level studies, socio-demographic factors (e.g., age, school or grade level) and environmental stressors or psychosocial factors (e.g., alcohol/drug use, examination workload, loneliness, low self-esteem, and victimization) have been found as determinants of anxiety-induced sleep disturbance among in-school adolescents [1,4,15,18,19,20,21].

Although country-level studies have identified the factors associated with anxiety disorders among in-school adolescents, there is a paucity of empirical analysis on this important subject in SSA in general. The scanty information on anxiety-induced sleep disturbance among in-school adolescents in SSA makes it difficult to design evidence-based policies and appropriate interventions to tackle this problem. This study, therefore, uses the Global School-based Health Survey of eleven countries between 2010 and 2017 to identify the factors that predict anxiety-induced sleep disturbance among in-school adolescents in SSA in order to fill the gap in research literature. Findings of the study will provide relevant information for policymakers and planners in the education sector to implement specific interventions and comprehensive plan to avert multiple risk factors and adverse effects of anxiety-induced sleep disturbance among in-school adolescents in SSA.

## 2. Materials and Methods

### 2.1. Study Design and Source of Data

This study involved a cross-sectional analysis of data from the Global School-Based Health Survey (GSHS) conducted among World Health Organization (WHO) countries. In the current study, we analyzed data from eleven countries in SSAwith a dataset between 2010 and 2017. The survey was conducted among in-school adolescents to examine health risk behaviors and protective factors using standardized questionnaires. The survey was collected nationwide in the selected countries with technical assistance from the WHO and the Centre for Disease Control and Prevention (CDC), in partnership with the Ministries of Health and Education. We relied on the “Strengthening the Reporting of Observational Studies in Epidemiology” (STROBE) statement in writing the manuscript.

### 2.2. Sampling Method

The survey used a two-stage sampling technique to select schools and students for the study. At the initial stage, schools were randomly selected based on probability proportionate to the school’s enrolment size. Secondly, the classes were selected randomly and all students aged 10–19 in the selected class who met the eligibility criteria were recruited for inclusion into the study. A total of 25,454 in-school adolescents from the 11 countries with complete cases of the variables of interest were included in the final analysis. A detailed description of the countries and their respective sample size has been shown in Table 1.

### 2.3. Study Variables

#### 2.3.1. Outcome Variable

The outcome variable was an anxiety-induced sleep disturbance. This was derived from the question “During the past 12 months, how often have you been so worried about something that you could not sleep at night?”. The responses were 1 = never, 2 = rarely, 3 = sometimes, 4 = most of the times, and 5 = always. The responses were dichotomized into Yes/No. Adolescents with response 1 = never, 2 = rarely and 3 = sometimes were classified as not having anxiety, denoted as “No”. Those who responded most of the times and always were classified as having anxiety-induced sleep disturbance (Yes). The dichotomization of the outcome variable was informed by the literature (e.g., [15,22]).

#### 2.3.2. Explanatory Variables

A total of 14 explanatory variables were used. The variables consisted of age, sex, truancy, bullying, marijuana use, peer support, close friends, suicidal ideation, suicidal plan, suicidal attempt, loneliness, parental or guardian supervision, parental or guardian bonding, and parental or guardian connectedness. All the explanatory variables were selected due to their significant association with an anxiety-induced sleep disturbance from literature (e.g., [15,22]) and availability from the datasets. The detailed description of the variables and the recoded responses have been shown in the Supplementary File attached.

### 2.4. Statistical Analyses

Data analysis was carried out using Stata software version 16.0 (Stata Corporation, College Station, TX, USA). Both descriptive and inferential analyses were performed. First, frequencies and percentages were used to present the results of categorical variables. Chart was used to present the prevalence of anxiety-induced sleep disturbance among in-school adolescents from the countries used. Regarding the inferential analysis, the Pearson chi-square test was first performed to examine the relationship between explanatory variables and the outcome variable (anxiety-induced sleep disturbance). The explanatory variables that showed statistical significance were placed in the second model (binary logistic regression) to determine the strength of the relationship. Two multivariable logistic regression models were built to determine the strength of the association between the outcome variable and the explanatory variables. Model I was fitted to determine the association between socio-demographic variables (i.e., age and sex) and anxiety-induced sleep disturbance. The second model (Model II) was fitted to determine the strength of the association between all the significant explanatory variables at the bivariate (chi-square analysis) and the outcome variable, adjusting for countries. The results of the regression analyses were presented using adjusted odds ratios (aOR) and their respective 95% confidence intervals (CIs). Statistical significance was set at *p*-value, *p* < 0.05 or 5% in all the inferential analyses. Additionally, a multicollinearity test was conducted using the Variance Inflation Factor (VIF). A mean VIF of 1.52 was found. Therefore, there was no evidence of multicollinearity among the variables used.

### 2.5. Ethical Consideration

Institutional permission was sought from either the Ministry of Health or the Ministry of Education in the selected countries. The survey was therefore conducted with adherence to the ethical guidelines by those institutions and for the conduct of studies among minors and majors. Written informed consent was obtained from the adolescents aged 18 years and above. For those below 18 years, a written parental or guardian consent and child assent were sought from each adolescent before inclusion in the study. The adolescents anonymously and voluntarily completed the survey.

## 3. Results

### 3.1. Prevalence of Anxiety-Induced Sleep Disturbance among In-School Adolescents in SSA

**Figure 1:**
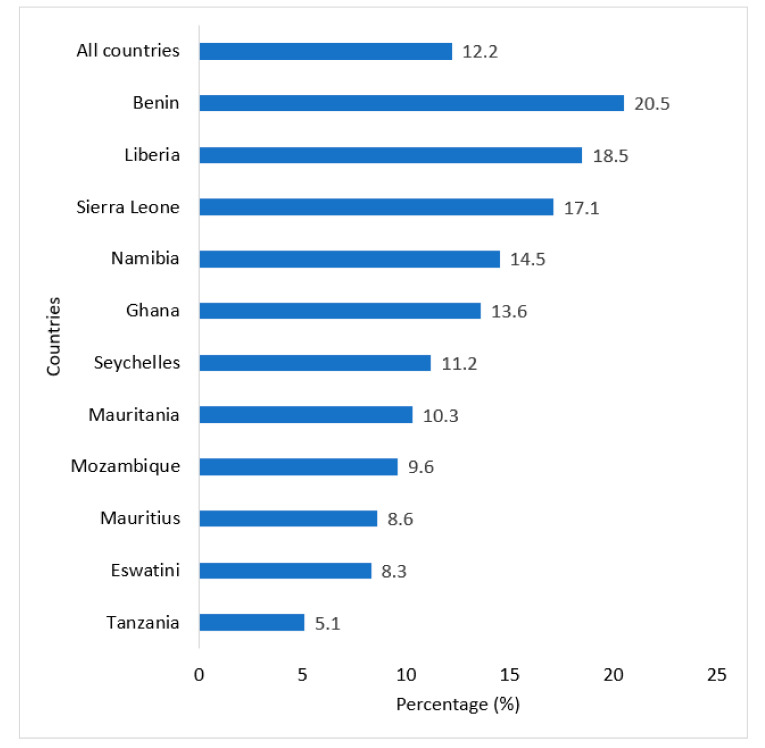
Prevalence of anxiety-induced sleep disturbance among in-school adolescents in sub-Saharan Africa.

The overall prevalence of anxiety-induced sleep disturbance among adolescents in SSA was 12.2%. The prevalence ranged from 5.1% in Tanzania to 20.5% in Benin as shown in Figure 1.

### 3.2. Prevalence of Anxiety-Induced Sleep Disturbance across the Background Characteristics of the Adolescents in SSA

Table 2 shows the results of the bivariate analysis of anxiety-induced sleep disturbance among in-school adolescents and the explanatory variables. The prevalence of anxiety-induced sleep disturbance among adolescents aged 15 and above was 13.5%. More females (12.8%) were found to be anxious. The prevalence of anxiety-induced sleep disturbance was high among adolescents who felt lonely (33.3%), those who were bullied (17.5%), those who had suicidal ideation (23.3%), those who had a suicidal plan (21.4%), those who attempted suicide (22.3%), those who currently use marijuana (22.0%), those who were truant at school (16.5%), those with no close friends (14.8%), and those with peer support (12.4%). In terms of the parental or guardian factors, the prevalence was higher among adolescents with no parental supervision (12.4%), those with no parental or guardian bonding (12.5%), and those with no parental connectedness (12.5%). The results from the chi-square test analysis showed that all explanatory variables except for peer support, parental or guardian supervision, and parental or guardian bonding were significantly associated with the anxiety-induced sleep disturbance. All the associated variables had a *p* < 0.05.

### 3.3. Predictors of Anxiety-Induced Sleep Disturbance among the In-School Adolescents in SSA

Table 3 presents the results of the binary logistic regression analysis. Age, sex, bullying, loneliness, suicidal behaviors (i.e., suicidal ideation, suicidal plan, and suicidal attempt), current marijuana use, school truancy, and the countries included in the study were the predictors of anxiety-induced sleep disturbance among in-school adolescents in SSA. The odds of an anxiety-induced sleep disturbance were higher among adolescents aged 15 and above [aOR = 1.26, 95% CI = 1.15, 1.39]. Male adolescents had lower odds of anxiety-induced sleep disturbance [aOR = 0.88, 95% CI = 0.81, 0.95] compared to their female counterparts. Additionally, the odds of an anxiety-induced sleep disturbance was higher among adolescents who were bullied [aOR = 1.54, 95% CI = 1.42, 1.67], felt lonely [aOR = 3.85, 95% CI = 3.52, 4.22], experienced suicidal ideations [aOR = 1.70, 95% CI = 1.52, 1.90], had suicidal plan [aOR = 1.26, 95% CI = 1.13, 1.41], made suicidal attempt [aOR = 1.21, 95% CI = 1.08, 1.35], used marijuana [aOR = 1.27, 95% CI = 1.06, 1.52], and were truant at school [aOR = 1.33, 95% CI = 1.22, 1.46].

## 4. Discussion

Anxiety has been linked to several adverse health events, yet existing studies in SSA have not prioritized this area. Thus, this study examined the prevalence and predictors of anxiety-induced sleep disturbance in SSA using the GSHS. It is evident from the findings that SSA has a relatively high prevalence of an anxiety-induced sleep disturbance. However, the prevalence of anxiety-induced sleep disturbance is not evenly distributed across the region as some countries have a higher prevalence rate than others. For instance, it was observed that anxiety-induced sleep disturbances were most prevalent in Benin (20.5%) and least prevalent in Tanzania (5.1%). The finding is supported by Dashiff et al. [23], and Osborn, Campbell, Weisz and Ndetei [24] who posit that anxiety disorders are likely to be elevated in SSA and other low- and middle-income countries (LMICs), compared to high-income countries like Mexico where anxiety disorders have been reported to be low [25]. The authors explained that the high levels of anxiety disorders among adolescents in SSA could be attributed to poverty, limited options for help seeking [23,24], and stigma around psychiatric syndromes detering help seeking. Other scholars also attribute the relatively high prevalence of anxiety-induced sleep disturbance among adolescents in SSA to high levels of stigma and mental disorders [26]. In this study, factors such as age, sex, loneliness, marijuana use, suicidal ideation, suicidal plan, truancy, and bullying were identified as the main correlates of anxiety-induced sleep disturbance among in-school adolescents in SSA.

Males had lower odds of anxiety-induced sleep disturbance compared to their female counterparts. This implies that in SSA, being a male could be a significant protective factor against anxiety-induced sleep disturbance. This observation mirrors a similar finding from an earlier study conducted by Remes, Brayne, van der Linde and Lafortune [27] that compared to males, females were twice likely to have anxiety disorders. Early studies show that females may be at elevated risk not only for disorder onset but also unfavorable course characteristics of anxiety (e.g., [28,29,30,31]). This sex variations in the likelihood of having anxiety-induced sleep disturbance is consistent in affirming an extant body of literature across varied spatio-temporal contexts (e.g., [32,33,34,35]). Likewise, the current finding has been re-echoed in some studies that established higher prevalence rates for females, suggesting higher levels of recurrence and/or persistence of anxiety disorders in girls and women (e.g., [16,27,30]). A possible explanation for why women are at higher risk of anxiety disorders compared to their male counterparts in SSA is that most females in SSA experience more adverse (e.g., domestic violence and abuse related setbacks) or other stressful life events (e.g., menstruation, pregnancy and childbirth), therefore exacerbating their odds of developing anxiety disorders [36,37].

Age was found as a significant factor associated with anxiety-induced sleep disturbance. Adolescents aged 15 or older had a higher likelihood of having anxiety-induced sleep disturbance, a finding that is congruent with previous studies (e.g., [36,38]) which identified age as a significant risk factor of anxiety disorders. Although adolescence may begin at age 10, substantial transitional changes (including physical, psychological, personality, cognitive, and attitudinal changes) occur after age 15 [39]. All of these changes are often characterized by heightened levels of anxiety. It is, therefore, not surprising that our findings observed higher odds of anxiety-induced sleep disturbance among adolescents aged 15 or older compared to younger adolescents. Current findings, however, contradict a myriad of studies that have concluded that persons within the younger age groups are more likely to have anxiety disorders [35,40,41,42,43]. For example, Madasu, Malhotra, and Kant et al. [43] found that adolescents aged 10–14 had a higher odds of anxiety-induced sleep disturbance compared with their counterparts aged 15–19. Context-specific and methodological variations in early studies may account for these inconsistencies.

Other results indicate that adolescents who have experienced bullying had higher odds of anxiety-induced sleep disturbance compared to those with no experience of bully. This is consistent with existing empirical findings [44,45]. Likewise, other studies have shown that there is a positive association between bullying victimization and the likelihood of anxiety disorders [46,47]. The justification for a higher likelihood of anxiety disorders among adolescents who have been victims of bullying could be due to the viewpoint that bullying victimization tends to lead to the internalization of symptoms. Hence, adolescents with such conditions are not identified on time and that intensifies their odds of anxiety disorders [48]. Another explanation for the high odds of anxiety-induced sleep disturbance among bullied adolescents is that bully victimization results in low self-esteem [49] which subsequently translates into anxiety disorders [50]. Such adolescents demonstrate high levels of anxiety when socializing with others [47].

Further, loneliness was identified as a significant factor associated with anxiety-induced sleep disturbance among in-school adolescents in SSA. This finding confirms the findings of other studies (e.g., [15,19,51]) that suggest association between anxiety disorders, be it generalized anxiety or social anxiety disorders, and truancy among adolescents. Adolescents who are confronted with parental occupational challenges and from low wealth quintile with less expenditure for family essentials may experience sporadic loneliness that could cause social anxiety. Similarly, adolescents’ loneliness can trigger an anxiety disorder because of inadequate peer support, and other significant others who usually play crucial roles in helping adolescents mitigate their potential stressful experiences [15,19]. This finding may be explained from another perspective that many truant adolescents display high levels of emotional distress, which when ill-managed, may result in anxiety disorders [52].

Adolescents who had suicidal ideation, suicidal plan or had attempted suicide had higher odds of anxiety-induced sleep disturbance [15,53,54]. The justification for this finding may be that, adolescents with suicidal ideation tend to have high levels of distress intolerance (inability to experience and withstand emotional distress) and emotional dysregulation which is all characteristic of anxiety disorders [55]. Alternatively, persons with a history of minor or major psychological, intrapersonal or individual-level factors and/or social problems (e.g., psychiatric complications, physical illness, and social disconnectedness with loved ones-parents, siblings) usually experience varying degrees of suicide ideations, plans and attempts [53,54,56]. These individuals usually experience different anxiety disorders such as generalized anxiety disorder, separation anxiety disorder, panic disorder, social phobia, specific phobia, obsessive-compulsive disorder and posttraumatic or acute stress disorder [3,12]. In addition, our finding confirms studies that have shown that anxious adolescents are more likely to attempt suicide [57]. 

Marijuana use was also identified as a factor associated with anxiety-induced sleep disturbance among in-school adolescents. Thus, adolescents who used marijuana were more likely to have anxiety-induced sleep disturbance compared to those who did not use it. This justifies the argument that marijuana use like tobacco and alcohol use are risk factors that increases an adolescent’s possibility of developing anxiety disorders [58]. It is a possible explanation that adolescents who are stressed tend to use marijuana to relieve themselves, and also prevent future anxiety and depression [59]. However, this dysfunctional and maladaptive coping attempt to relieve stress triggers anxiety disorders as well as the other negative effects cited earlier [60].

### Limitations and Implications

The dataset used in this study adopted cross-sectional design and as such, current findings did not indicate any causal relations. Also, the dataset used was self-reported and therefore, prone to recall and social desirability biases that are beyond the control of the research team. Again, the findings may not be applicable to out-of-school adolescents since the data used captured only the responses of in-school adolescents. Moreover, the use of a secondary dataset limited the analysis to only variables within the dataset. Hence, important factors such as the experience of an adverse life event [36], that has been identified to potentially increase the risk of anxiety-induced sleep disturbance among adolescents was not available and could not be assessed. We also used a single question from the GSHS to measure anxiety-induced sleep disturbance, which may potentially obscure specific and/or priming effects associated with specific anxiety disorders [22,61]. Additionally, the assessment of anxiety relied on a simple self-report question pertaining to “worry” that may be influenced by several factors such as personality. As such, conclusions and inferences from our findings must be considered in light of the limitations aforementioned.

Despite the limitations, the findings are quintessential to policy and practice. As anxiety-induced sleep disturbance usually takes progressive, persistent, or recurrent trajectories, it is critically required to examine not only risk factors but also predictors of anxiety in order to identify high-risk adolescents for developmental challenges for targeted early interventions (e.g., Rational Emotive Behavioral Education, [REBE], Social Emotional Learning [SEL] and policy realignment (e.g., early school-based screening). The findings of the study emphasize the need for the governments in SSA to formulate national policies and frameworks that will be targeted specifically to the sub-populations who are at higher risks of anxiety-induced sleep disturbance. As part of the measures to reduce the prevalence of anxiety in SSA, there is the need to prioritize the school setting because of its contextual influences on anxiety-induced sleep disturbance. Practical measures (e.g., marijuana cessation therapy, face-face counseling) can also help reduce the use of marijuana among in-school adolescents as a means of reducing the prevalence of anxiety-induced sleep disturbance.

## 5. Conclusions

The study extends what is known about the prevalence and predictors of anxiety-induced sleep disturbance among in-school adolescents in SSA. Overall, SSA has a relatively high prevalence of anxiety anxiety-induced sleep disturbance among in-school adolescents. However, the prevalence rate varies substantially across the studied countries. In-school adolescents who are at most risks of anxiety-induced sleep disturbance include female adolescents, those who currently use marijuana, those who feel lonely, those who had suicidal ideations, a suicidal plan and had made suicidal attempts, as well as those who are truant at school. These findings, therefore, highlight the need for policies and interventions that target in-school adolescents within the most at-risk populations of anxiety-induced sleep disturbance in SSA. Further studies could adopt a qualitative approach to bring to the fore the reasons and facilitate deeper understanding of anxiety among in-school adolescents in SSA. Also, a comparative analysis of anxiety disorder among in-school and out-of-school adolescents can be conducted in future studies to deepen the discourse on anxiety-induced sleep disturbance in SSA.

## Figures and Tables

**Table 1:** Description of the study sample.

Country	Year of Publication	Population	Sample ^a^	Percentage
Benin	2016	2536	2219	8.7
Eswatini	2013	3680	2944	11.6
Ghana	2012	3632	2821	11.1
Liberia	2017	2744	1499	5.9
Mauritania	2010	2063	1531	6.0
Mauritius	2017	3012	2491	9.8
Mozambique	2015	1918	1319	5.2
Namibia	2013	4531	3525	13.8
Seychelles	2015	2540	1891	7.4
Sierra Leone	2017	2798	2118	8.3
Tanzania	2014	3793	3096	12.2
**All countries**		33,247	25,454	100.0

Sample ^a^ = Sample with complete cases of variables used in the study.

**Table 2:** Anxiety-induced Sleep Disturbance among In-school Adolescents in SSA by Explanatory Variables.

	*n* = 25,454		Anxiety-Induced Sleep Disturbance		
**Variables**	**Frequency**	**Percentage**	**No (%)**	**Yes (%)**	***p*-Value**
**Age**					<0.001
14 years or younger	8222	32.3	90.5	9.5	
15 years or older	17,232	67.7	86.5	13.5	
**Sex**					0.001
Female	13,153	51.7	87.2	12.8	
Male	12,301	48.3	88.5	11.5	
**Felt lonely**					<0.001
No	22,273	87.5	90.8	9.2	
Yes	3181	12.5	66.7	33.3	
**Bullied**					<0.001
No	15,579	61.2	91.2	8.8	
Yes	9875	38.8	82.5	17.5	
**Suicidal ideation**					<0.001
No	21,323	83.8	90.0	10.0	
Yes	4131	16.2	76.7	23.3	
**Suicidal plan**					<0.001
No	20,904	82.1	89.8	10.2	
Yes	4550	17.9	78.6	21.4	
**Suicidal attempt**					<0.001
No	21,261	83.5	89.8	10.2	
Yes	4193	16.5	77.8	22.3	
**Current marijuana use**					<0.001
No	24,508	96.3	88.2	11.8	
Yes	946	3.7	78.0	22.0	
**Truancy**					<0.001
No	18,694	73.4	89.4	10.6	
Yes	6760	26.6	83.5	16.5	
**Close friends**					<0.001
No	2581	10.1	85.2	14.8	
Yes	22,873	89.9	88.1	11.9	
**Peer support**					0.533
No	17,310	68.0	87.9	12.1	
Yes	8144	32.0	87.6	12.4	
**Parental or guardian supervision**					0.234
No	14,519	57.0	87.6	12.4	
Yes	10,935	43.0	88.1	11.9	
**Parental or guardian connectedness**					0.042
No	15,215	59.8	87.5	12.5	
Yes	10,239	40.2	88.3	11.7	
**Parental or guardian bonding**					0.058
No	15,690	61.6	87.5	12.5	
Yes	9764	38.4	88.3	11.7	

Source: GSHS, 2010–2017 Note: Pearson chi-square test was used to obtain *p*-values.

**Table 3:** Multivariable regression analysis of predictors of anxiety-induced sleep disturbance among in-school adolescents in SSA.

Variable	Model I	Model II
	aOR [95% CI]	aOR [95% CI]
**Age**		
14 years or younger	Ref.	Ref.
15 years or older	1.50 *** [1.37, 1.63]	1.26 *** [1.15, 1.39]
**Sex**		
Female	Ref.	Ref.
Male	0.86 *** [0.80, 0.93]	0.88 ** [0.81, 0.95]
**Bullied**		
No		Ref.
Yes		1.54 *** [1.42, 1.67]
**Felt lonely**		
No		Ref.
Yes		3.85 *** [3.52, 4.22]
**Suicidal ideation**		
No		Ref.
Yes		1.70 *** [1.52, 1.90]
**Suicidal plan**		
No		Ref.
Yes		1.26 *** [1.13, 1.41]
**Suicidal attempt**		
No		Ref.
Yes		1.21 ** [1.08, 1.35]
**Current marijuana use**		
No		Ref.
Yes		1.27 * [1.06, 1.52]
**Truancy**		
No		Ref.
Yes		1.33 *** [1.22, 1.46]
**Close friends**		
No		Ref.
Yes		0.95 [0.84, 1.07]
**Parental or guardian connectedness**		
No		Ref.
Yes		1.05 [0.97, 1.15]
N	25,454	25,454
Pseudo R ^2^	0.0053	0.1149

AOR = Adjusted Odds Ratio; CI = Confidence Interval, Ref = Reference category, N = Sample size, Model II = Adjusted for countries. * *p* < 0.05, ** *p* < 0.01, *** *p* < 0.001.

## Data Availability

The dataset is available freely at https://www.who.int/ncds/surveillance/gshs/datasets/en/ (accessed on 18 February 2021).

## Supplementary Materials

The following are available online at https://www.mdpi.com/2227-9032/9/2/234/s1, Table S1: Study variables.
