# Supplementary material for: A Multi-Country Analysis of Prevalence of Anxiety-Induced Sleep Disturbance and Its Associated Factors among In-School Adolescents in Sub-Saharan Africa Using the Global School-Based Health Survey
Source: Healthcare (Basel). 2021 Feb 22;9(2):234. doi: 10.3390/healthcare9020234 (PMC7926929; doi:10.3390/healthcare9020234)
Supplement: Supplementary file 1 [file healthcare-09-00234-s001.pdf]

Table 1. Study variables.

**Variables**	**Question**	**Response options and recoding**
**Outcome variable**		
Anxiety-induced sleep disturbance	During the past 12 months, how often have you been so worried about something that you could not sleep at night?	1 = never to 5 = always (coded 1 - 3 = 0, 4 – 5 = 1**)**
**Explanatory variables**		
Socio-demographic characteristics		
Age	How old are you?	1=12, 2=13, 3=14, 4=15, 5=16, 6=17, 7=18 years (coded as 0=1-14, 15-19)
Sex	What is your sex?	1=male, 2=female (coded 2=0, 1=male)
Psychosocial environmental factors		
Marijuana use	During the past 30 days, how many times have you used marijuana (Country specific examples)	1=0 times; to 5=20 or more times (coded as 1=0; and 2-5=1)
Loneliness	During the past 12 months, how often have you felt lonely?	1=never, 2=rarely, 3= sometimes, 4 = most of the time to 5 = always (coded as 1-3 = 0; and 4- 5 = 1)
Truancy	During the past 30 days, on how many days did you miss classes or school without permission?	1=0 days,2= 1or 2 days, 3=3 to 5 days, 4=6 to 9 days, 5= 10 or more (coded as 1=0; and 2-5=1)
Suicidal ideation	During the past 12 months, did you ever seriously consider attempting suicide?”	1 = yes, 2 = no (coded 2 = 0; and 1 = 1)
Suicidal attempt	During the past 12 months, how many times did you actually attempt suicide?	1=0 times; to 5= 6 or more times (coded as 1=0; and 2-5=1)
Suicidal plan	During the past 12 months, did you make a plan about how you would attempt suicide?	1 = yes, 2 = no (coded 2 = 0; and 1 = 1)
Bullied	During the past 30 days, how were you bullied most often?	1 = 0 times; to 8 = 12 or more times (coded as 1 = 0; and 2–7 = 1)
Close friends	How many close friends do you have?	1=0 to 4=3 or more (coded as 1=0; and 2-4=1)
Helpful (Peer support)	During the past 30 days, how often were most of the students in your school kind and helpful?	1=never, 2=Rarely, 3=sometimes, 4=most of the times, 5=always (coded as 1-3 = 0; and 4–5 = 1)
Parents check homework (parental supervision)	During the past 30 days, how often did your parents or guardians check to see if your homework was done?	1=never, 2=Rarely, 3=sometimes, 4=most of the times, 5=always (coded as 1-3 = 0; and 4–5 = 1)
Understand problems (Parental Connectedness	During the past 30 days, how often did your parents or guardians understand your problems and worries?	1=never, 2=Rarely, 3=sometimes, 4=most of the times, 5=always (coded as 1-3 = 0; and 4–5 = 1)
Know what adolescent do free	During the past 30 days, how often did your parents or guardians really know what you were doing with your free time?	1=never, 2=Rarely, 3=sometimes, 4=most of the times, 5=always (coded as 1-3= 0; and 4–5 = 1)

time (Parental or guardian Bonding)		
